# The Phytoplankton Taxon-Dependent Oil Response and Its Microbiome: Correlation but Not Causation

**DOI:** 10.3389/fmicb.2019.00385

**Published:** 2019-03-11

**Authors:** Tatiana Severin, Deana L. Erdner

**Affiliations:** Marine Science Institute, The University of Texas at Austin, Port Aransas, TX, United States

**Keywords:** phytoplankton, bacteria, interaction, oil, dispersant

## Abstract

Phytoplankton strongly interact with their associated bacteria, both attached (PA), and free-living (FL), and bacterial community structures can be specific to phytoplankton species. Similarly, responses to environmental stressors can vary by taxon, as exemplified by observed shifts in phytoplankton community structure from diatoms to phytoflagellates after the Deepwater Horizon (DWH) oil spill. Here, we assess the extent to which associated bacteria influence the phytoplankton taxon-specific oil response by exposing xenic and axenic strains of three phytoplankton species to oil and/or dispersant. The dinoflagellates *Amphidinium carterae* and *Peridinium sociale*, and the diatom *Skeletonema* sp., all harbored significantly distinct bacterial communities that reflected their host oil response. Oil degrading bacteria were detected in both PA and FL communities of the oil resistant dinoflagellates, but their FL bacteria were more efficient in lipid hydrolysis, a proxy for oil degradation capability. Inversely, the growth rate and photosynthetic parameters of the diatom *Skeletonema* sp. was the most impacted by dispersed oil compared to the dinoflagellates, and oil-degrading bacteria were not significantly associated to its microbiome, even in the dispersed oil treatment. Moreover, the FL bacteria of *Skeletonema* did not show significant oil degradation. Yet, the lack of consistent significant differences in growth or photosynthetic parameters between the xenic and axenic cultures after oil exposure suggest that, physiologically, the associated bacteria do not modify the phytoplankton oil response. Instead, both oil resistance and phycosphere composition appear to be species-specific characteristics that are not causally linked. This study explores one aspect of what is undoubtedly a complex suite of interactions between phytoplankton and their associated bacteria; future analyses would benefit from studies of genes and metabolites that mediate algal-bacterial exchanges.

## Introduction

Phytoplankton are important contributors to carbon dioxide fixation (Field, 1998), biogeochemical cycling, and aquatic food webs. The response of phytoplankton to anthropogenic disturbances, like oil pollution, remains difficult to predict and often varies in a taxon-dependent manner. The Deepwater Horizon (DWH) oil spill in April 2010 was the largest accidental oil spill in United States history, affecting the Gulf of Mexico coast from Louisiana to Florida (Sammarco et al., 2013). In the aftermath of DWH, phytoplankton abundance declined and community shifts were observed (Parsons et al., 2015), with responses that varied according to the phytoplankton species (González et al., 2009; Ozhan et al., 2014a; Fiori et al., 2016) and their surface:volume ratio (Echeveste et al., 2010). Most studies noted a decline in diatom biomass and an increase in phytoflagellates after exposure to oil in both environmental (Fiori et al., 2016) and laboratory settings (Harrison et al., 1986; Mishamandani et al., 2015), but some observed the opposite (González et al., 2009; Gilde and Pinckney, 2012; Parsons et al., 2015). These equivocal results highlight that the taxon-specific phytoplankton oil response is complex and depends on several factors: oil type and concentration (Dunstan et al., 1975; Huang et al., 2011; Ozhan et al., 2014a), nutrient concentrations (Ozhan and Bargu, 2014), initial phytoplankton community structure (Prouse et al., 1976), zooplankton oil tolerance (González et al., 2009), and phytoplankton-associated bacteria (Mishamandani et al., 2015; Severin et al., 2016; Thompson et al., 2017).

Strong interactions exist between phytoplankton and their associated bacteria, the so-called phycosphere, which comprises both phytoplankton-attached (PA) and free-living (FL) bacteria (Cole, 1982). These interactions are often beneficial for both organisms. For example, bacteria can promote phytoplankton growth or increase their production of secondary metabolites (Amin et al., 2015; Palacios et al., 2016). In turn, phytoplankton release strain-dependent organic compounds (Becker et al., 2014) utilized by specific bacteria, leading to host-specific bacterial community structures (Bagatini et al., 2014). Recent studies have shown the presence of oil-degrading bacteria in some phycospheres (Mishamandani et al., 2015; Severin et al., 2016; Thompson et al., 2017) that can enhance hydrocarbon degradation (Abed, 2010).

Considering these observations, this study aimed to determine if the phytoplankton taxon-specific bacterial community plays a role in the phytoplankton species-dependent oil and/or dispersant response. Three phytoplankton were selected for their varying oil resistance, in order from most to least resistant as determined from preliminary oil exposure experiments (data not shown): *A. carterae*, a non-armored dinoflagellate; *P. sociale*, an armored dinoflagellate; and *Skeletonema* sp., a small diatom. The specific strains used here were isolated from the Gulf of Mexico (Kooistra et al., 2008; Aké-Castillo and Vásquez, 2011; Licea et al., 2013), the source of the Light Louisiana sweet crude oil used on this study. The 3 species were made axenic to differentiate the contribution of the microbiome from that of the phytoplankton cell. The different phytoplankton oil responses were studied in both xenic and axenic strains in terms of growth rate and photophysiology, as they are indicators of the capacity for carbon fixation and production, and therefore have implications for ecosystem function after an oil spill. The comparison of the xenic (with phycosphere) and axenic (without phycosphere) cultures exposed to undispersed and dispersed oil revealed that their oil response was inherent to the phytoplankton itself and not significantly affected by the phycosphere. Nevertheless, the species-specific microbiomes matched their host oil responses. Unlike the diatom phycosphere, several oil degrading bacterial taxa were found in the dinoflagellate microbiomes, and their ability to efficiently degrade oil corresponded with *Amphidinium* and *P*. resistance to dispersed oil.

## Materials and Methods

### Phytoplankton Cultures and Axenization

An armored dinoflagellate, *P. sociale* (UTEX LB 1948), and a non-armored dinoflagellate, *A. carterae* (UTEX LB 1561) were obtained from the UTEX culture collection of algae of the University of Texas at Austin. *Skeletonema* sp. was isolated from the eastern Gulf of Mexico, USA (Tyre, 2018). Cultures were maintained in sterile f/2 medium (salinity of 32) (Guillard, 1975) and incubated at 25°C under a 12:12 light:dark cycle at an irradiance of ~100 μmol photons m^−2^ s^−1^.

Axenic strains of *P. sociale, A. carterae* and *Skeletonema* were created using a protocol adapted from Su et al. (2007) and Azma et al. (2010). Aliquots of exponential phase culture (6 replicates of 15 mL each) were washed 5 times by centrifugation at 1,000 × g for 10 min. The supernatants were discarded, and the cell pellets were resuspended in 10 mL sterile 0.2 μm-filtered seawater. The remaining attached bacteria were detached with a protocol adapted from Velji and Albright (1993). Tetrasodium pyrophosphate (PPi) was added to a final concentration of 0.2 mM, and the samples were incubated 30 min in the dark at ambient temperature with shaking at 120 rpm. The PPi optimal concentration, the incubation time, and the use of shaking rather than sonication were determined prior to the experiment. The samples were then washed 5 times by centrifugation at 1,000 × g for 10 min, supernatants were discarded, and cell pellets were resuspended in 10 mL sterile 0.2 μm-filtered seawater. After the fifth centrifugation, supernatants were discarded, and cell pellets were resuspended in 5 mL sterile f/2 medium containing different antibiotic mixtures. The successful antibiotic combinations were 250 μg mL^−1^ amoxicillin and 250 μg mL^−1^ ciprofloxacin (ciprofloxacin stock solution at pH 1 for a better dissolution) for *P. sociale*, amoxicillin 500 μg mL^−1^ and ciprofloxacin 500 μg mL^−1^ (ciprofloxacin stock solution at pH 6) for *A. carterae*, and amoxicillin 300 μg mL^−1^ and neomycin 1 mg mL^−1^ for *Skeletonema* sp. The treated cultures grew for 4 weeks in these conditions between transfers and required 3 to 5 transfers into fresh sterile f/2 medium with their respective antibiotics before they were axenic.

The presence of bacteria was assessed by epifluorescence microscopy using 4′,6-diamidino-2-phenylindolestain (DAPI) staining. Glutaraldehyde was added to 1 mL sample at a final concentration of 1% followed by 15 min incubation in the dark at ambient temperature. DAPI was added to a final concentration of 1 μg L^−1^ followed by incubation in the dark for 15 min at ambient temperature. Cells were filtered onto a 0.2 μm black polycarbonate membrane (Whatman, USA) and immediately examined by epifluorescence microscopy (Olympus BX41, 400x magnification). Bacterial presence was also assessed by flow cytometry as described below.

### Experimental Design

Xenic (X) and axenic (AX) cultures of *A. carterae, P. sociale*, and *Skeletonema* sp. were exposed to 4 different treatments in triplicate. Dispersant treatments (D) contained 0.5 ppm of COREXIT 9,500, oil treatments (O) contained 10 ppm of light Louisiana sweet crude oil, and dispersed oil treatments (OD) contained 0.5 ppm of dispersant COREXIT 9,500 and 10 ppm of oil (1:20 dispersant:oil). No additions were made to control cultures (C). The 10 ppm oil concentration was chosen based on previous studies (Ozhan et al., 2014b and references therein) and preliminary tests examining phytoplankton sublethal effects. Approximately 1,000 cells mL^−1^ of X and AX *A. carterae, P. sociale*, and *Skeletonema* sp. in mid-log phase were inoculated into 500 mL Erlenmeyer flasks containing 500 mL of 0.2 μm filtered and sterilized f/2 medium (salinity of 32). All experimental flasks were incubated at 25°C under a 12:12 light:dark cycle at an irradiance of ~100 μmol photons m^−2^ s^−1^.

### Phytoplankton and Bacteria Abundances

For *A. carterae* and *Skeletonema* sp. abundance, 1.5 mL of sample were collected every 24 h, fixed for 15 min at ambient temperature with 1% glutaraldehyde (final concentration) and stored at −20°C until analyses on a Becton Dickinson Accuri C6 flow cytometer using phytoplankton natural fluorescence (Olsen et al., 1993). For *P. sociale*, 1.5 mL of sample was collected every 24 h, preserved with Lugol's iodine solution at 1% final concentration (Wetzel and Likens, 1990) and stored in the dark at ambient temperature. The abundance of *P. sociale* was determined via microscopy under brightfield illumination using a Sedgewick Rafter counting chamber. Phytoplankton growth rate (μ, d^−1^) was calculated during exponential growth according to the following equation:

$$\begin{array}{l} \mu =\;\frac{\ln\left(C2\right)-\ln\left(C1\right)}{\text{t}2-\text{t}1} \end{array}$$

where C_1_ and C_2_ are the cell concentrations (cells ml^−1^) at time 1 (t_1_, d) and time 2 (t_2_, d), respectively.

To determine bacterial abundance, 10 mL of sample were collected every 24 h from each flask, fixed for 15 min at ambient temperature with 1% glutaraldehyde (final concentration) and stored at −20°C. Before flow cytometry analyses, samples were thawed and split in 2 sub-samples. For enumeration of free-living (FL) bacteria, 1 mL was filtered through a 3 μm polycarbonate filter (Whatman, USA) to remove algal cells with phytoplankton-attached bacteria (PA), leaving only FL bacteria in the filtrate. A second aliquot of 1 mL was treated with PPi (final concentration 0.2 mM) and incubated 30 min in the dark at ambient temperature with shaking at 120 rpm to release the attached bacteria from their host. This sample contained both the PA (now detached) + FL bacteria. The 2 sub-samples (FL only and PA + FL) were stained with SYBR Green I (Invitrogen, USA) at a final concentration of 10^−4^ (v/v) for 15 min at ambient temperature in the dark, before enumeration of the bacteria using a Becton Dickinson Accuri C6 flow cytometer according to the method described in Mével et al. (2008). PA bacterial abundances were calculated as the difference between PA + FL abundance and FL only abundance.

### Photosynthetic Parameters

Every 24 h, 10 mL of sample were collected from each flask and placed in the dark for 30 min. The photosynthetic efficiency of photosystem II (Fv/Fm; dimensionless) and the photosystem II (PSII) cross section (Sigma PSII in nm^−2^; or physical area occupied by PSII) were measured by Fast Repetition Rate fluorometry (FRRf) on a Fast^TRACKA^ instrument (Chelsea Technologies Group Ltd.), which was also used to determine the potential maximum relative electron transport rate (potential rETRmax; dimensionless) and the efficiency of photosynthesis (alpha; dimensionless) by Rapid Light Curve measurements (RLC). The FRRf was conducted with an acquisition of 100 saturation flashes of 1 μs duration with an interval of 1 μs between pulses. Twelve sequences were performed per acquisition with an interval of 100 ms between each sequence. The same protocol was used for the RLC acquisition, with PAR steps from 0 to ~600 μmol photon m^−2^ s^−1^. RLC data were analyzed in R software (version 3.4.1) using the package phytotools.

### Bacterial Extracellular Enzymatic Activities

Extracellular enzymatic activities, specifically aminopeptidase, β-glucosidase, and lipase, were measured every 2 days (Hoppe, 1983). The <3 μm fraction was collected for measurement of FL bacterial enzymatic activity in the X cultures, and as a check for contamination in the AX cultures. Unfiltered samples were used for measurement of total activity, consisting of phytoplankton activity only in the AX cultures, and phytoplankton + FL + PA bacterial activities in the X cultures. Triplicate subsamples (390 μL) were incubated 4 h in the dark at 25°C with L-leucine-7amido-4-methyl coumarin (L-LA, 6.25 μM final concentration), 4-methylumbelliferyl-β-D-glucoside (β-Glc, 6.25 μM final concentration), or 4-methylumbelliferyl-palmitate (Lip, 1.25 μM final concentration) (Wambeke et al., 2009). These saturating concentrations for L-LA, β-Glc and Lip and optimized incubation times were determined from previous experiments. For each enzyme measured, a blank was prepared using sterile 0.2 μm filtered seawater incubated in the same conditions as the samples. Aminopeptidase, β-glucosidase, and lipase incubations were stopped by addition of 50 μL of SDS 10%, glycine-ammonium hydroxide buffer (0.05 M glycine + 0.2 M NH_4_OH, pH10.5), or Tris 1M pH 11, respectively. Samples were then stored at −20°C until fluorescence measurement on a spectrofluorometer, after the addition of 550 μl of borate buffer (pH 10, Sigma-Aldrich).

The PA bacterial enzyme activity in the X cultures was determined by difference after measuring the activity of the whole X sample (FL bacteria + PA bacteria + phytoplankton). The activity of the FL fraction was subtracted from that of the total fraction to obtain the activity of the phytoplankton + PA bacteria. Activity measured in the attached fraction of the AX cultures (> 3 μm) was considered as phytoplanktonic extracellular enzymatic activity; this AX cell-specific phytoplankton activity was subtracted from the activity of the phytoplankton + PA bacteria of the X cultures to obtain the PA bacterial extracellular enzyme activities. Finally, aminopeptidase, glucosidase, and lipase activities were normalized by FL and PA bacteria abundances to obtain the bacterial cell-specific extracellular enzyme activities.

Extracellular enzyme activities of the PA (EEA_PA_) and FL (EEA_FL_) bacteria (mol.L^−1^.h^−1^.cell^−1^) were calculated according to the following equations:

$$\begin{array}{l} \text{EE}{\text{A}}_{\text{PA}}=\frac{f_{totalx}-f_{FLx}-\left(\frac{f_{PAax}}{P_{ax}}\times P_{x}\right)}{a\times t}/B_{PA}\\ \text{EE}{\text{A}}_{\text{FL}}=\frac{f_{FLx}}{a\times t}/B_{FL} \end{array}$$

Where f_totalx_ is the fluorescence in the total fraction of the X cultures, f_FLx_ is the fluorescence in the FL fraction of the X cultures and f_PAax_ is the fluorescence of the PA fraction in the AX cultures. P_ax_ and P_x_ are the phytoplankton abundances (cell.L^−1^) in the AX and X cultures, respectively. B_PA_ and B_FL_ are the bacteria abundances (cells.L^−1^) in, respectively, the PA and FL fractions of the X cultures. a (mole^−1^) is the slope of the fluorophore calibration curves, and t (h) is the incubation time (here 4 h).

Fluorescence in the live samples were corrected using the blank of the corresponding enzyme activity. In cases where enzyme activity (fluorescence) was low, negative values sometimes arose from the correction due to blank variability. These small negative values were replaced by null values. Previous studies used filtered and unfiltered samples to measure the extracellular enzyme activities in different bacterial fraction (Crump et al., 1998; Karrasch et al., 2003), which reinforce the method of measurements and calculations used in our study. Moreover, we are more interested here in the pattern of extracellular enzyme activities for each bacterial fraction than in the absolute values, that can be quite different from environmental studies.

### DNA Extraction and 16S rRNA Gene Sequencing

Samples for DNA extraction were collected at the beginning of the experiment for the controls (C.T0), and from each flask of all treatments at the end of the exponential phase (7 days for *A. carterae* and *P. sociale* and 6 days for *Skeletonema* sp.; C.TF). About 300 mL of culture were sequentially filtered through 3 μm (PA bacteria fraction) and 0.2 μm (FL bacteria fraction) Whatman polycarbonate filters. DNA was extracted from the filters using the AllPrep mini kit (Qiagen). DNA samples from *Skeletonema* sp. were sequenced at the Research and Testing Laboratory (Lubbock, TX), all others were sequenced at the Research Technology Support Facility at Michigan State University. The V4 hypervariable region of the bacterial 16S rRNA gene was amplified using the primers 515F (5′-GTGYCAGCMGCCGCGGTAA-3′) and 806R (5′-GGACTACNVGGGTWTCTAAT-3′) and sequenced on a MiSeq platform to generate 2 × 250 bp paired-end reads. Paired-end sequences were stitched, and the resulting ~300 bp fragments were filtered using QIIME 1.9.1 (Caporaso et al., 2010), with chimeras removed using UCHIME. The cleaned sequences were clustered into OTUs at a distance of 0.03, assigned using the Greengenes database (McDonald et al., 2012), and filtered to remove Archaea, mitochondria, and chloroplast OTUs. To enable comparison between samples, sequences were randomly re-sampled (Lozupone et al., 2011) from the filtered OTU table to the same depth as the sample with the fewest bacterial sequences (6,751 reads) using Mersenne twister PRNG (QIIME 1.9.1). All further analyses were performed on the resulting rarefied OTU table. For multivariate statistical analyses, OTU abundance was averaged between triplicates.

### Bacterial Community Analyses

Non-parametric species richness estimator Chao1 and Shannon diversity index were calculated using the R package phyloseq (McMurdie and Holmes, 2013). Both indices were used because they capture different aspects of community composition. Chao1 is based on relative abundance, thus it primarily measures richness, while the Shannon index considers both richness and evenness. Principal Coordinate analyses (PCoAs; R package phyloseq) based on Bray-Curtis similarity were generated for the bacterial communities of each phytoplankton species, and for all species together. Significant bacterial clusters were identified in the multi-species PCoA via an analysis of similarities (ANOSIM; R package vegan). Similarity percentage (SIMPER, 999 iterations, R package vegan) was then used to determine which individual OTUs contributed most to the dissimilarity between these significant bacterial clusters. Indicator Species analysis (R package indicspecies; Cáceres et al., 2010) was used to identify the OTUs significantly correlated with significant clusters identified in the single-species PCoA via ANOSIM. The results were visualized as networks with the igraph R package (Csárdi and Nepusz, 2006). Canonical Correspondence Analysis (CCA) was performed with the R package vegan to investigate the parameters influencing the bacterial community structures: phytoplankton growth rate and photophysiology, phytoplankton exudates (aminopeptidase and glucosidase activities), or the different treatments (oil and/or dispersant and lipase activity). These parameters were transformed according to their pairwise distributions. Prior to CCA, Spearman's rank pairwise correlations were calculated between all transformed variables for each culture separately and all together. As the presence of correlated variables in the same CCA would lead to superimposed arrows, only one variable was selected and the covariables were removed from the analyses to get a clearer picture (Ter Braak and Smilauer, 2002). A summary of the correlated variables and the corresponding selected one for the CCAs is in Table S4. A forward selection was then conducted (R package vegan) to select the significant non-correlated variables (*p*-values < 0.05), while maximizing the explained variation.

### Statistical Analysis of Physiological Parameters

ANOVA (R package stats) were performed on X and AX phytoplankton growth rates, the averaged X and AX photosynthetic parameters, and on the averaged FL and PA extracellular enzyme activities to determine the significance of each treatment compared to the control. Dunnett's *post-hoc* tests were then conducted between the control and each treatment. In each treatment, differences between X and AX phytoplankton parameters (growth rate and photophysiology), and between FL and PA bacterial extracellular enzyme activities were analyzed using student tests. The photosynthetic parameters Fv/Fm, SigmaPSII, and potential rETRmax were averaged over the time to simplify data visualization and because the time was not a significant variable (*p*-values > 0.05) when a Linear Mixed-effects Model was fitted (LMM; R package lme4). The bacterial abundances were not averaged over the time because of a significant increase over the time (LMM, *p*-values > 0.05 for FL and PA bacteria of each phytoplankton species). LMM was fitted to the FL and PA bacterial abundances separately using all time points and was followed by a Dunnett *post-hoc* test to determine the differences between each treatment and the control. The differences between FL and PA bacterial abundances in each treatment were analyzed using an LMM and a Tukey *post-hoc* test using the R package emmeans.

## Results

### Phytoplankton Oil Responses

**Figure 1:**
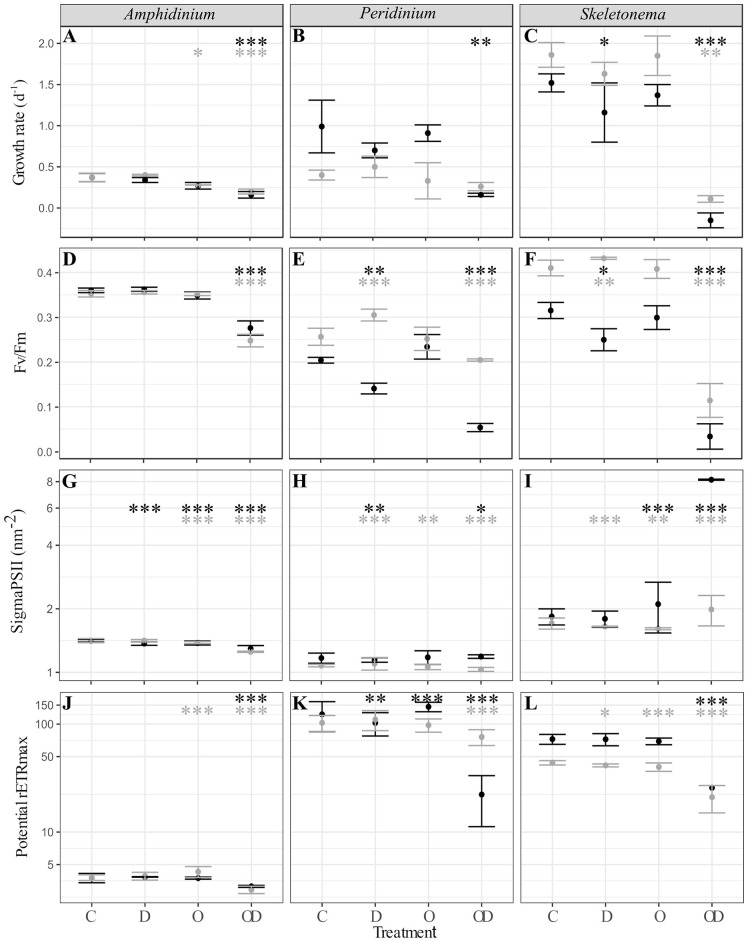
**(A–C)** Phytoplankton growth rate (d^−1^); **(D–F)** photosynthesis efficiency (Fv/Fm, dimensionless); **(G–I)** PSII cross section (sigmaPSII, nm^−2^); and **(J–L)** potential maximal relative electron transport rate (potential rETRmax, dimensionless) of xenic (black) and axenic (gray) cultures of **(A,D,G,J)** *A. carterae*; **(B,E,H,K)** *P. sociale*; and **(C,F,I,L)** *Skeletonema* sp. Treatments are C for control, D for dispersant alone, O for crude oil, OD for dispersed crude oil. The photosynthetic parameters were averaged over time and the error bars represent the standard deviation between the triplicates. Stars represent the significant Dunnett's test *p*-values (ANOVA *post-hoc* tests) comparing the control to the other treatments for the xenic (black) and axenic (gray) cultures. *p < 0.05; **p < 0.01; ***p < 0.001. *P*-values resulting from pairwise students tests between X and AX physiological parameters for each culture are in Table S1.

A comparison between all the treatments showed that compared to control (C), dispersed oil (OD) had the greatest impact (Figure 1), resulting in significant decreases in all physiological measures of both X and AX cultures *A. carterae* (noted AmphX and AmphAX hereafter), *P. sociale* (noted PeriX and PeriAX hereafter), and *Skeletonema* sp. (noted SkelX and SkelAX hereafter), with two exceptions. Growth rate of PeriAX showed no significant difference from control, and photosystem II cross section (Sigma PSII) of SkelX significantly increased relative to control. Compared to control, growth rates of AmphX, PeriX, and SkelX were reduced by 57, 84, and 106%, respectively, while photosynthetic efficiency (Fv/Fm) decreased by 23, 74, and 89%, respectively. Similar results were observed in AmphAX, PeriAX, and SkelAX, where growth was reduced by 45, 32, and 94%, respectively, and Fv/Fm decreased by 30, 20, and 72%, respectively. Then, in both X and AX cultures, the dispersed oil treatment had the greatest physiological impacts on *Skeletonema* sp. compared to the control, while *A. carterae* and *P. sociale* were the least impacted amongst the X and AX cultures, respectively. SkelX physiology was also significantly affected by the dispersant alone, with a growth rate decrease of 38% and a Fv/Fm decrease of 20% compared to control.

Comparison between X and AX cultures showed that differences in growth rate and photophysiological responses between X and AX cultures to dispersed oil varied by species, without any consistent pattern (*p*-values of Student tests in Table S1). No significant difference in growth rate, Fv/Fm, sigma PSII, or potential maximal relative electron transport rate (potential rETRmax) were observed between AmphX and AmphAX cultures, except in the dispersed oil treatment where the photosynthetic parameters were significantly higher in AmphX (student tests, *p*-values < 0.001). PeriX cultures had higher growth rate and sigma PSII (student tests, *p*-values < 0.05 and < 0.001 for the growth rate and sigma PSII, respectively), except in the dispersed oil where PeriAX growth was significantly faster (student test, *p*-value = 0.03). In contrast, AmphAX, PeriAX, and SkelAX showed significantly higher Fv/Fm (student tests, *p*-values < 0.001) except with oil alone. SkelAX growth rate and Fv/Fm were significantly higher than SkelX in all treatments (student tests, *p*-values < 0.05), but the inverse was observed for sigma PSII and potential rETRmax (student tests, *p*-values < 0.001).

### Bacterial Oil Response

**Figure 2:**
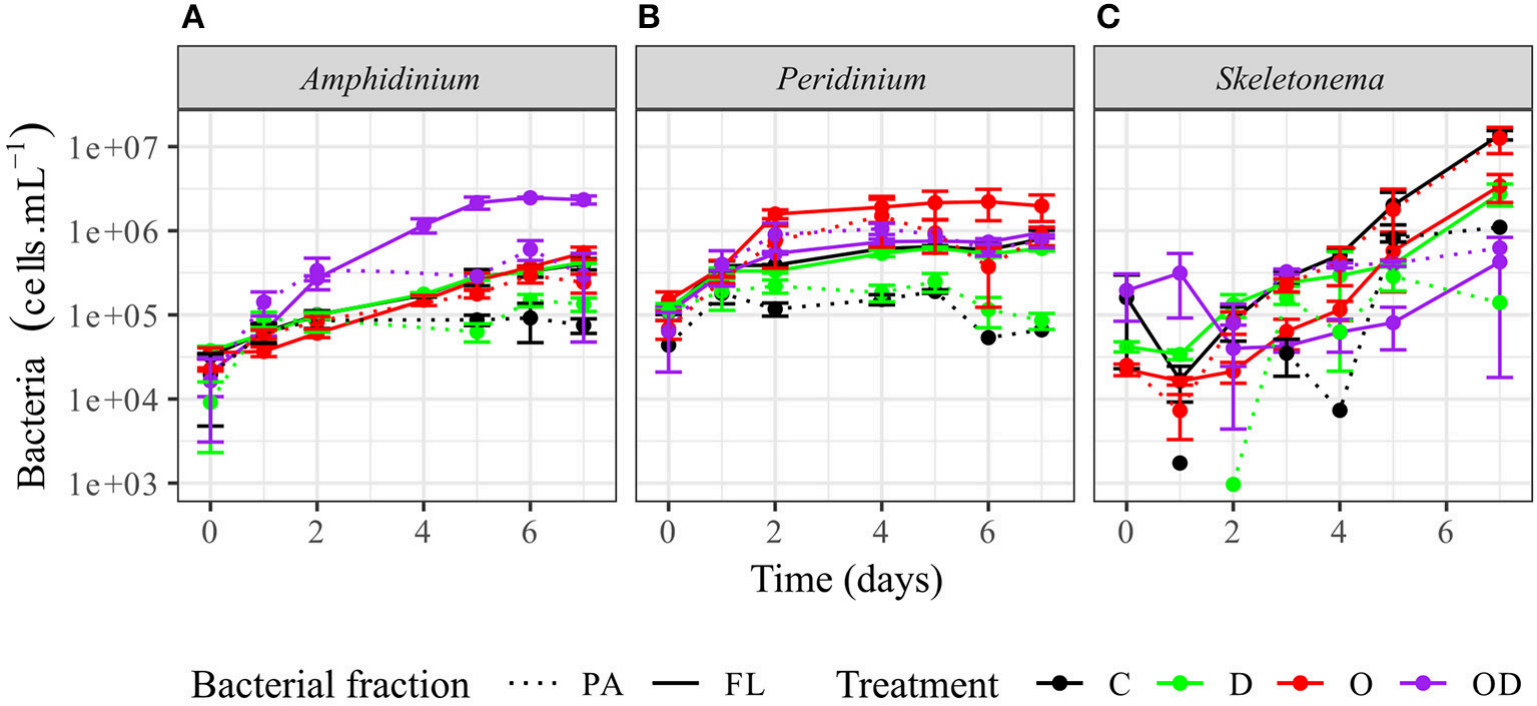
Abundance of the phytoplankton-attached (PA, dashed line) and free-living (FL, solid line) bacteria in **(A)** *A. carterae*, **(B)** *P. sociale*, and **(C)** *Skeletonema* sp. cultures for each treatment. Controls are shown in black, dispersant alone in green, oil alone in red, and dispersed oil in purple. The error bars represent the standard deviation of the triplicates.

FL bacteria were significantly more abundant than PA bacteria in AmphX, PeriX, and SkelX(LMM *post-hoc* Tukey test; Figure 2; Table S2), except in SkelX oil treatments where FL and PA bacteria abundances were not significantly different. Addition of dispersed oil or crude oil alone significantly affected bacterial abundances of the dinoflagellates. Both FL and PA bacterial abundances significantly increased in AmphX and PeriX in response to dispersed and undispersed oil, respectively (Table S2). In the dinoflagellate cultures, FL bacteria in the oil treatment and PA bacteria in the dispersed oil treatment reached the same abundance as the FL bacteria in the control. PA and FL bacteria of SkelX in the dispersed oil treatment remained low or significantly decreased compared to the control (~5 × 10^5^ cells mL^−1^) while PA bacteria in the oil treatment significantly increased 11-fold (Table S2).

**Figure 3:**
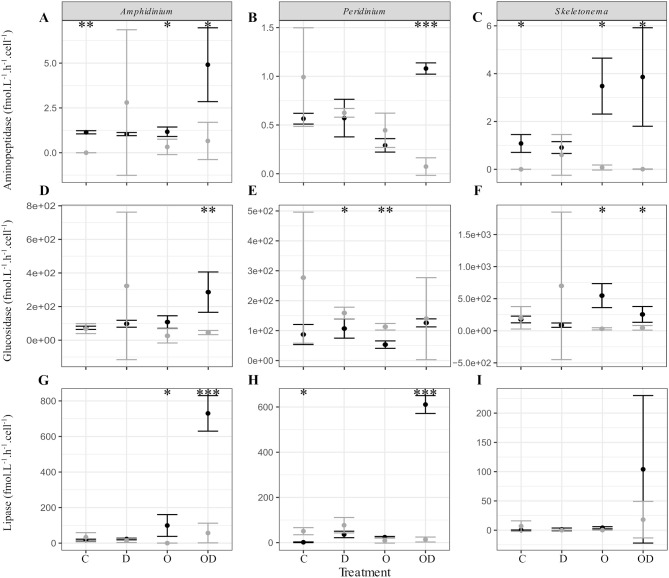
**(A–C)** Aminopeptidase; **(D–F)** glucosidase; and **(G–I)** lipase extracellular activities (fmol.L^−1^.h^−1^.cell^−1^) of the free-living (black) and phytoplankton -attached (gray) bacteria in X cultures of **(A,D,G)** *A. carterae*; **(B,E,H)** *P. sociale*; and **(C,F,I)** *Skeletonema* sp. Treatments are C for control, D for dispersant alone, O for crude oil, OD for dispersed crude oil. The extracellular activities were averaged over time. The error bars represent the standard deviation of the triplicates. Stars indicate the *p*-value of pairwise Student tests between FL and PA extracellular activities. *p < 0.05; **p < 0.01; ***p < 0.001. Results of Dunnett tests (ANOVA *post-hoc* tests) between the control and the treatments in each phytoplankton culture are in Table S2.

We further explored the capacity of the associated FL and PA bacteria to degrade phytoplankton-derived organic matter via cell-specific aminopeptidase and glucosidase extracellular activities measurements, as well as their capacity to degrade dispersed and undispersed oil with the measurement of lipase extracellular activity (Ziervogel et al., 2012). In general, no significant differences were observed between the control and the treatments for the PA bacteria. For the FL bacteria, high lipase activity indicated that AmphX and PeriX FL microbiomes were able to degrade dispersed oil, but SkelX microbiome was not (Figures 3G–I; Table S2). Dispersed oil also significantly increased the aminopeptidase extracellular activities of the FL bacteria of both dinoflagellates, and the glucosidase activity of only AmphX FL bacteria, compared to the control (Table S2) and to the PA bacteria (Figure 3). Undispersed oil was the only treatment that significantly increased the glucosidase activity of SkelX FL bacteria (Table S2), which was significantly higher than the PA bacteria, likewise for aminopeptidase. In general, the *Skeletonema* microbiome had a higher aminopeptidase and glucosidase activities than the dinoflagellate microbiomes.

### Bacterial Community Structures Across Phytoplankton Species

**Figure 4:**
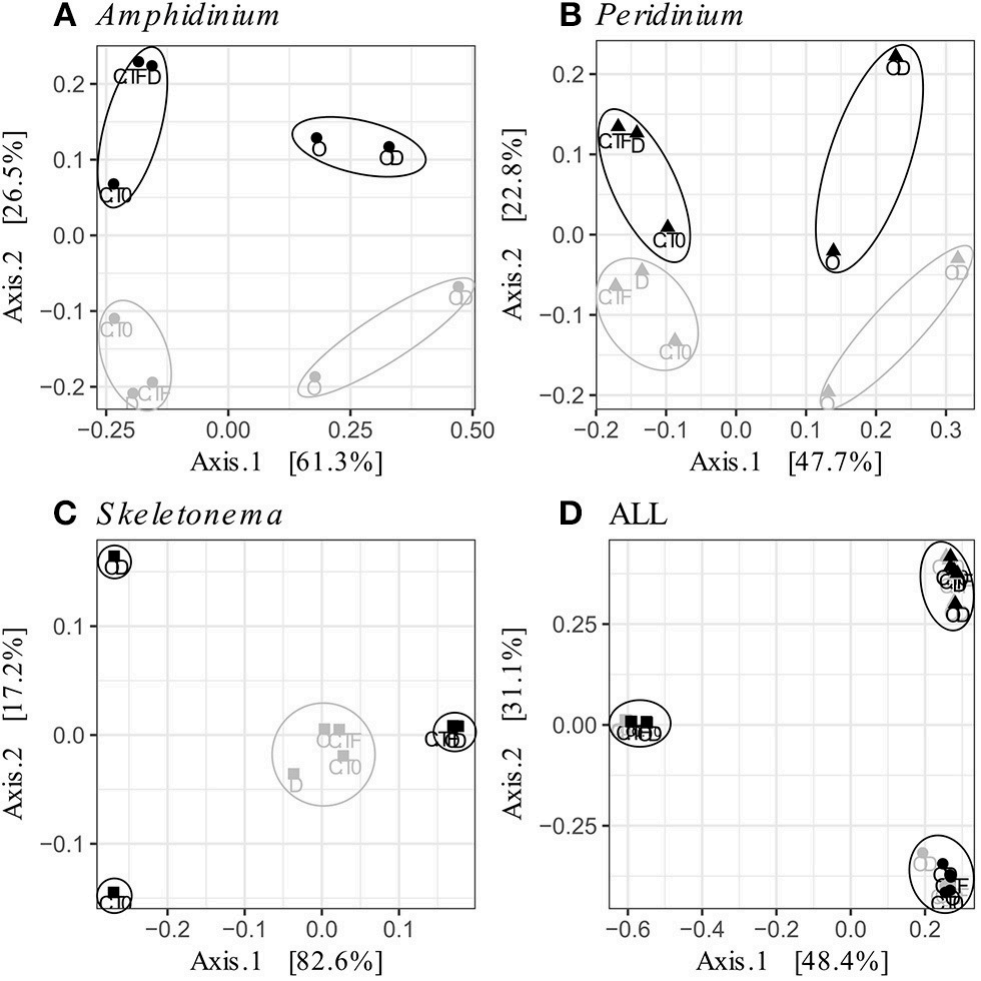
Principal Coordinate Analysis (PCoA) based on Bray-Curtis dissimilarity of the phytoplankton-attached (gray) and the free-living (black) bacterial communities for each phytoplankton species: **(A)** *A. carterae* (circles), **(B)** *P. sociale* (triangles), **(C)** *Skeletonema* sp. (squares), and **(D)** all species together. Labels are the treatments: CT0 for control sampled at time 0, CTF for control sampled at final time point; D for dispersant, O for oil, and OD for dispersed oil all at final time point. The circles represent the significant clusters as determined by ANOSIM.

**Figure 5:**
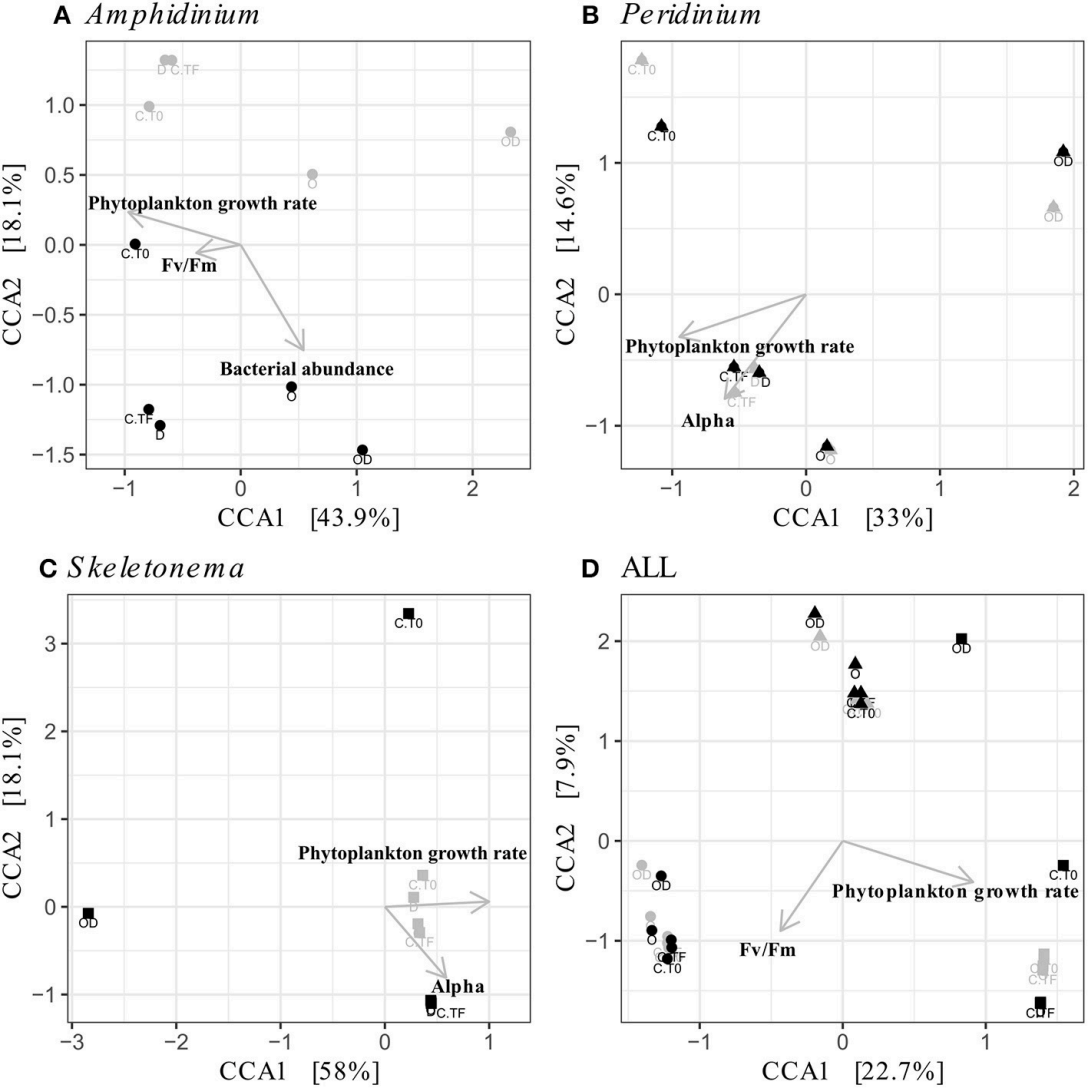
Canonical Correspondence Analyses (CCA) of the phytoplankton-attached (PA, gray) and the free-living (FL, black) bacterial communities for each phytoplankton species: *A. carterae* (**A**, circles), *P. sociale* (**B**, triangles), *Skeletonema* sp. (**C**, squares), and all species together **(D)**. Labels are the treatments: CT0 for control sampled at time 0, CTF for control sampled at final time point; D for dispersant, O for oil, and OD for dispersed oil all at final time point.

FL and PA bacteria community structures were significantly different between phytoplankton species (ANOSIM test, R = 0.99, *p*-value < 0.001; Figure 4D). The AmphX microbiome was characterized by the presence of *Alcanivorax* (12.37% average) and *Hyphomicrobiaceae* (11.79% average), which were found at 0–0.1% in the other species (Table S3). The SkelX microbiome was dominated by *Flavobacteriaceae* (75.85%) and *Pyruvatibacter* (15.40%), which were 0–0.45% in the dinoflagellate cultures. Lastly, there was a different *Flavobacteriaceae* OTU that characterized PeriX (17.40%) yet was only 0–0.03% in the other two species. A small number of OTUs also distinguished the two dinoflagellate species from each other, specifically two *Marivita* OTUs that were more abundant (6.22 and 10.53%) in AmphX than in PeriX (0.02 and 0.07%), and *Flavobacteraceae* (11.47%) and *Rhodobacteraceae* (7.25%) OTUs in PeriX that were only 0.01–0.02% in AmphX. SkelX microbiome comprised relatively few OTUs (19.08 ± 3.32) compared to that of AmphX (178.55 ± 23.29) and PeriX (149.71 ± 11.20), and had significantly lower richness and diversity (Chao1 = 31.53 ± 15.83, Shannon = 0.64 ± 0.29; Table 1) compared to AmphX and PeriX microbiome (Chao1 = 218.57 ± 29.88 and 191.25 ± 27.29, Shannon index = 3.31 ± 0.25 and 3.13 ± 0.19, respectively). CCA (Figure 5D) showed that 30.6% of the variance was explained by the phytoplankton growth rate and photophysiology, with SkelX bacterial communities associated with high phytoplankton growth rate and AmphX microbiomes with high Fv/Fm.

**Table 1:** Number of observed OTUs (Operational Taxon Units), richness estimator Chao1 and Shannon diversity index at 0.03 level of clustering of the re-sampled bacterial OTU tables, for the free-living (FL) and phytoplankton-attached (PA) bacteria of each phytoplankton species: *A. carterae, P. sociale*, and *Skeletonema* sp.

		***Amphidinium***		***Peridinium***		***Skeletonema***	
		**FL**	**PA**	**FL**	**PA**	**FL**	**PA**
Observed OTUs	C.T0	174.00 ± 9.16	160.67 ± 12.05	151.00 ± 10.53	157.00 ± 10.15	21.33 ± 2.08	21.67 ± 0.58
	C.Tf	186.00 ± 9.54	210.33 ± 50.81	144.67 ± 0.58	157.33 ± 3.05	17.33 ± 0.58	20.67 ± 4.04
	D	194.00 ± 7.07	177.67 ± 8.50	156.00 ± 5.66	162.33 ± 2.08	18.67 ± 3.51	21.50 ± 3.53
	O	189.33 ± 7.02	173.00 ± 7.81	132.00 ± 15.56	145.33 ± 10.97	14.33 ± 1.15	19.33 ± 1.15
	OD	174.00 ± 6.24	151.67 ± 27.54	143.70 ± 4.51	144.00 ± 16.00	17.67 ± 5.13	NA
Chao1	C.T0	198.97 ± 12.83	196.18 ± 10.12	201.50 ± 26.82	216.53 ± 52.62	45.92 ± 27.98	25.80 ± 3.17
	C.Tf	226.29 ± 15.01	261.95 ± 74.55	189.72 ± 23.96	200.29 ± 16.47	27.83 ± 11.25	33.84 ± 18.64
	D	238.00 ± 11.31	214.29 ± 7.54	192.55 ± 10.26	202.82 ± 6.82	50.00 ± 21.38	30.67 ± 3.30
	O	228.63 ± 15.37	206.52 ± 12.65	165.39 ± 29.37	178.53 ± 21.33	18.83 ± 5.96	28.00 ± 3.60
	OD	218.90 ± 9.39	202.40 ± 20.92	180.82 ± 18.64	176.15 ± 39.03	22.58 ± 8.50	NA
Shannon	C.T0	3.50 ± 0.04	3.15 ± 0.19	3.22 ± 0.04	3.26 ± 0.08	1.19 ± 0.01	0.80 ± 0.06
	C.Tf	3.55 ± 0.10	3.49 ± 0.18	3.16 ± 0.04	3.19 ± 0.02	0.32 ± 0.04	0.66 ± 0.02
	D	3.73 ± 0.00	3.31 ± 0.00	3.23 ± 0.05	3.28 ± 0.01	0.33 ± 0.08	0.78 ± 0.09
	O	3.30 ± 0.05	3.09 ± 0.11	2.72 ± 0.17	2.83 ± 0.11	0.32 ± 0.05	0.69 ± 0.01
	OD	3.09 ± 0.05	2.98 ± 0.18	3.25 ± 0.02	3.04 ± 0.09	0.67 ± 0.24	NA

*Treatments are CT0 for control sampled at time 0, CTF for control sampled at final time point; D for dispersant, O for oil, and OD for dispersed oil all at final time point. NA for not available data*.

### Bacterial Community Structures Within Each Phytoplankton Species

**Figure 6:**
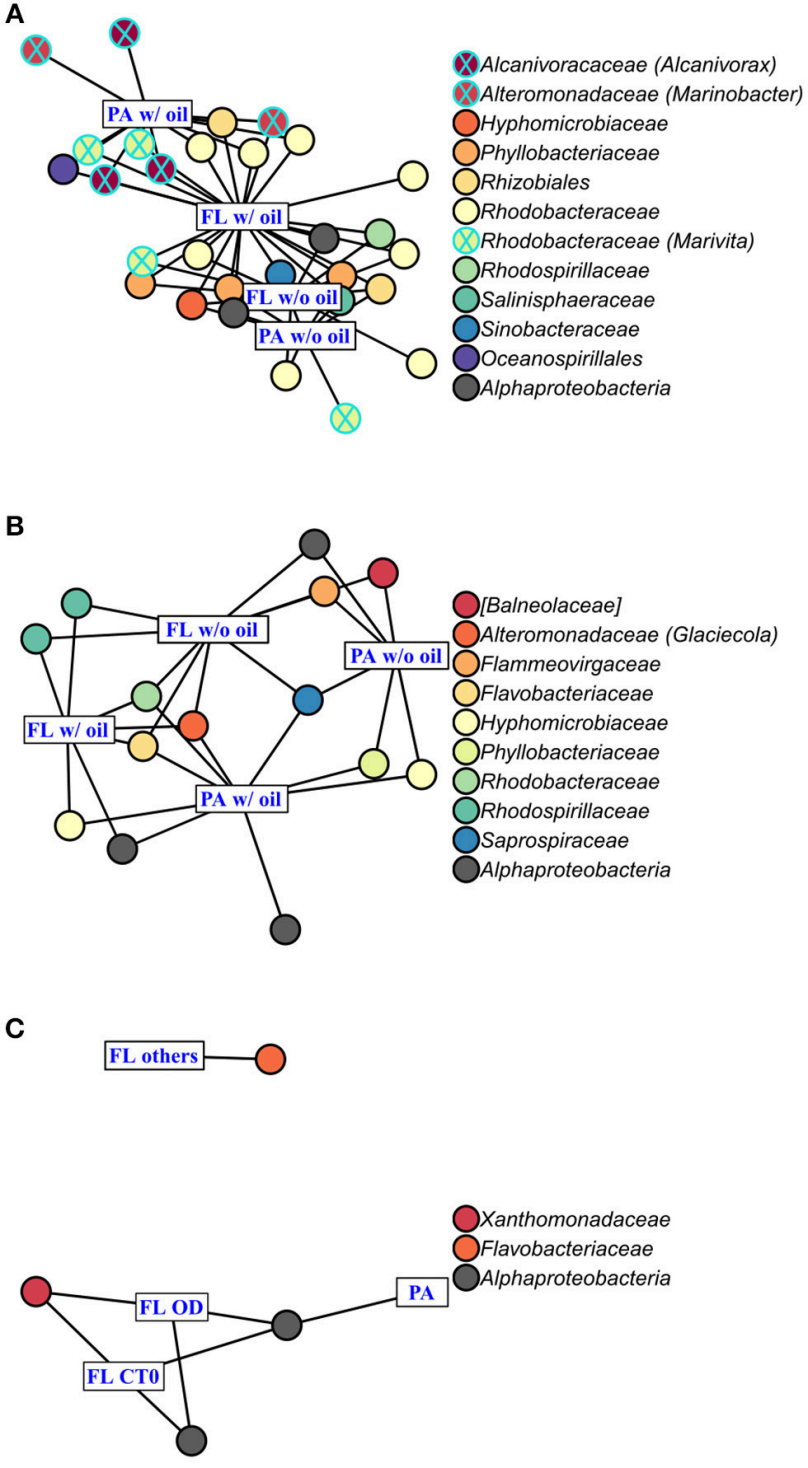
Indicator Species Analysis of each significant cluster identified by PCoA (Figure 4) and ANOSIM, for **(A)** *A. carterae*, **(B)** *P. sociale*, and **(C)** *Skeletonema* sp. microbiomes. Rectangles represent the treatment clusters annotated in blue, where “FL others” in **(C)** includes the CTf, D and O treatments. Circles are the significant indicator OTUs (see Table S2 for the detailed Indicator Species Analysis results), the fill colors show OTU taxonomy at the family level with the genus into bracket when assigned. Known oil-degrading OTUs are circled in blue, and non-oil-degrading OTUs are circled in black.

AmphX and PeriX microbiomes showed similar patterns with four significantly different clusters segregating the FL and PA bacterial communities in the treatments with oil (dispersed and undispersed) and without oil (controls and dispersant) (Figures 4A,B; ANOSIM tests, R = 0.89 and 0.67, *p*-values = 4 × 10^−4^ and 0.0016 for AmphX and PeriX, respectively). While AmphX FL and PA microbiomes exposed to dispersed and undispersed oil were characterized by known-oil degraders, *Alcanivorax* and *Marinobacter* (Figure 6A, Figure S1; Table S5), no known oil-degraders were significantly associated to PeriX microbiomes exposed to dispersed and undispersed oil (Figure 6B, Figure S1; Table S5). Several *Marinobacter* and *Marivita* OTUs, known oil-degraders, were present in PeriX microbiome, but were not specific to the oil treatments. Similarly, *Marivita* was present and significantly associated to AmphX FL and PA microbiomes not exposed to oil (Figure 6A, Figure S1; Table S5). AmphX microbiomes exposed to oil were significantly constrained by high bacterial abundance, whereas in PeriXcultures they were associated with low phytoplankton growth rate and photosynthetic efficiency (Figures 5A,B; Table S4). The dinoflagellate microbiomes that were not exposed to oil were significantly correlated with phytoplankton physiology: high phytoplankton growth rate and Fv/Fm (Table S4) for both PeriX and AmphX.

SkelX microbiomes separated into four significantly different clusters (Figure 4C; ANOSIM test, R = 1, *p*-value = 0.0015). The PA microbiomes clustered together, regardless of treatment. They were characterized by an *Alphaproteobacteria* shared with the FL communities from C.T0 and the OD treatments, each of which formed its own cluster (Figure 6C; Table S5). No known oil-degraders were significantly correlated with any SkelX microbiome cluster. The FL bacterial community structures in the OD treatment and control (T0) clusters were significantly correlated with low phytoplankton growth rate and photophysiology (Figure 5C) and the presence of dispersant (Table S4).

OTUs representing known oil-degraders were found amongst the microbiomes of all species. In SkelX, a unique OTU assigned as the known oil degrader *Alteromonas* accounted for 30% of the total reads of the FL bacteria exposed to dispersed oil, but only 0.01% of the PA bacteria treated with dispersant only, and 0.005% of the FL bacteria exposed to dispersant only (Figure S1). The abundance of this *Alteromonas* OTU did not drive community similarity, however, as the SkelX FL microbiomes split between 3 distinct clusters (Figure 4). Meanwhile, AmphX FL bacterial communities exposed to dispersed oil were composed of 51 OTUs reported as oil degraders that accounted for 50.7% of the total reads of this sample and were also present in the controls in lower relative abundance. AmphX FL bacteria exposed to undispersed oil, and PA bacteria exposed to undispersed and dispersed oil, showed similar values: 47, 52 and 47 oil-degrader OTUs, accounting for 51.9, 60.8, and 57.2% of the total reads in these samples, respectively. Similarly, PeriX FL bacteria exposed to undispersed or dispersed oil, and PA bacteria exposed to undispersed or dispersed oil, comprised 26, 26, 27, and 25 oil-degrader OTUs, accounting for 31.8, 25.0, 36.0, and 29.5% of the total reads in these samples, respectively.

## Discussion

### Minor Influence of the Phycosphere on the Phytoplankton Taxon-Specific Oil Response

The similar phytoplankton oil response in the presence and absence of the phycosphere was not expected. The strong phytoplankton-bacteria interactions highlighted in numerous studies (Cole, 1982; Amin et al., 2012; Durham et al., 2017), and the detection of oil-degrading bacteria in phytoplankton phycospheres (Severin et al., 2016; Thompson et al., 2017) suggests that dispersed and undispersed oil would be more deleterious to axenic phytoplankton. Here, growth rate and photosynthetic efficiency of axenic cultures of *P. sociale* and *Skeletonema* sp. were significantly higher than those of the xenic cultures when exposed to dispersed oil (Figure 1; Table S1). This indicates that the phytoplankton oil response is less driven by its species-specific phycosphere than by its own physiology. One caveat is that the oil response of axenic cultures, and algal physiology in general, could be influenced by the time since removal of their phycosphere. Here the cultures were made axenic only 4 months before the experiments. Uribe and Espejo (2003) showed that xenic and freshly axenic (100 days) cultures of *Alexandrium catenella* had similar growth rates. Similarly, Jauzein et al. (2015) showed that an axenic culture of *Alexandrium tamarense* inoculated with bacteria from its xenic clone required more than a year to regain physiological rates similar to the xenic parental strain. Thus, the physiological influence of the phycosphere may persist for some period after bacterial removal, and likewise be slow to return after bacterial addition. Because of this, it may be necessary to maintain strains in the axenic condition for a long period to fully return to the algal “baseline” physiology and responses. To detect specific responses, global analyses like comparative transcriptomics might highlight specific processes affected by the absence of bacteria, observable at the molecular level but not with more integrated measures like growth rate and photophysiology.

Despite the lack of a consistent difference between xenic and axenic pairs, our study revealed that the 3 phytoplankton species have significantly different oil responses (Figure 1). The diatom *Skeletonema* sp. was more sensitive to dispersed oil than the dinoflagellates, while *A. carterae* was more resistant than *P. sociale* (Figures 1A–F); sensitivity being defined here as the amplitude of their physiological response (growth rate and photosynthesis efficiency) to a similar stimulus. These observations are in accordance with previous studies showing a higher oil sensitivity of small centric diatoms compared to flagellates or pennate diatoms (Echeveste et al., 2010; Fiori et al., 2016). None of the phytoplankton were impacted by crude oil alone, which agrees with previous studies showing that phytoplankton are more sensitive to dispersed oil than to crude oil alone (Hsiao et al., 1978; Jung et al., 2012; Severin et al., 2016), because dispersant promotes greater release of water-soluble hydrocarbon compounds (Fiocco and Lewis, 1999). Dispersed oil also resulted in a significant increase in PSII cross-section in xenic *Skeletonema* sp. (sigma PSII; Figure 1I), as it tried unsuccessfully to maintain its photosynthetic efficiency. This is in accordance with previous studies that reported a stimulation of chlorophyll synthesis in response to photosynthetic inhibition by different pollutants (Fiori and Pistocchi, 2014; Fiori et al., 2016). These responses suggest that the phytoplankton growth during an oil spill should not be monitored using chlorophyll fluorescence, because of the ability of some species to modify their chlorophyll and PSII content.

### Dinoflagellate Phycospheres Contain Oil-Degrading Taxa

Despite the lack of significant differences in growth and photosynthetic function between the axenic and xenic phytoplankton, the oil responses of the associated bacteria in the xenic cultures matched their host oil resistance. The greatest changes in activity were observed in the FL bacterial communities, which were more abundant (Figure 2) and showed higher lipase activity when exposed to dispersed oil (proxy of oil degradation; Figure 3; Ziervogel et al., 2012). This suggests the development of oil degraders only in the dinoflagellate FL bacteria communities, similar to a previous study on *Heterocapsa* sp. (Severin et al., 2016), where oil degraders were observed only in the FL fraction, but in contrast to the results of Thompson et al. (2017) who observed higher oil degradation activity in the PA fraction. Composition of the communities also changed, as evidenced by 16S rRNA gene sequences (Figures 4A,B, Figure S1). Oil-exposed FL and PA communities were characterized by lower richness and diversity (Table 1) and by the development of known oil degraders like the alkane-degrading *Alcanivorax, Marinobacter*, and *Marivita* (Gutierrez et al., 2013; Kimes et al., 2014; Figure 6A). In contrast to a previous study on *Heterocapsa* sp. (Severin et al., 2016), here the PA bacterial community exposed to oil is not resilient, as it is more similar to the oil-treated FL bacterial community than the PA controls.

The physical association of the PA bacteria with the algal cells does not prevent the community changes even though they presumably have stronger and more direct interactions with the phytoplankton cells compared to the FL bacteria. The PA bacteria do not seem to have greater exchange with phytoplankton than the FL bacteria (Simon, 1985; Ziervogel et al., 2010; Rieck et al., 2015), as evidenced by similar cell-specific FL and PA aminopeptidase and glucosidase activities (Ziervogel et al., 2012; Figures 3A,B,D,E). In *A. carterae*, the PA community was driven by the phytoplankton growth rate (Figure 5A), suggesting that the dinoflagellate still influences its attached bacterial community through interactions that appear to involve processes other than the provision of aminopeptides and glucose. In contrast, the FL bacterial community was not impacted by the dinoflagellates, but rather by crude oil and elevated bacterial abundances (Table S4). Moreover, in the absence of the bacterial abundance, the CCA of *A. carterae* microbiome was not significant, highlighting the minor influence of the dinoflagellate on its FL bacteria in the presence of oil. This is not true for *Skeletonema* cultures (Table S4), where the FL bacteria in the dispersed oil treatment are negatively correlated with growth rates of their host and by high concentration of oil (Figure 5C).

### *Skeletonema* Phycosphere Lacks Oil Degradation Activity

The oil response of *Skeletonema* sp. bacterial communities was notably different from that of the dinoflagellate phycospheres. In the oil and dispersed oil treatments, neither the FL bacterial abundance (Figure 2) nor the cellular lipase activity (Figure 3) increased. This is corroborated by the almost complete lack of oil degraders in *Skeletonema* sp. phycosphere determined by 16S rRNA gene sequencing. Community structure in the diatom phycosphere was strongly related to the phytoplankton physiology and its exudates, not to oil exposure (Figure 5C; Table S4). Only one OTU, assigned as the known polycyclic aromatic hydrocarbon-degrading bacteria *Alteromonas* (Gutierrez et al., 2013), was observed, and only in the FL bacterial community exposed to dispersed oil (Figure 4, Figure S1) yet was not indicative of dispersed oil (Figure 6C). Moreover, unlike the dinoflagellate phycospheres, no oil-degraders were observed in *Skeletonema* sp. controls. Oil treatment did result in increased bacterial abundance in the PA fraction (Figure 2C; Table S2), but this seems to be due to bacteria attached to aggregates and not to phytoplankton, as there is no phytoplankton growth that could support more attached bacteria (Figure 1C). This is in accordance with previous studies that observed the formation of marine oil snow (MOS) in both oil-polluted environments (Passow et al., 2012) and laboratory experiments (van Eenennaam et al., 2016). It also has been shown that stressed phytoplankton, for example after oil exposure, can increase their production of extracellular polymeric substances (Xiao and Zheng, 2016), a compound required for MOS formation.

Overall, the bacterial community structures observed between the three phytoplankton species show wide variation in structure and function. Of the taxon examined, only *Skeletonema* sp. microbiomes lack oil degradation capabilities, which is congruent with their different community structure i.e., the absence of known oil-degrading taxa. The two dinoflagellate microbiomes, however, have distinct community structures, even though they share a similar oil response in terms of degradation capabilities and the emergence of OTUs associated with oil degradation. These differences in structure are maintained even after oil exposure, which indicates that the increase in abundance of similar oil-degrading taxa is not sufficient to mask the specificity of their microbiomes. The presence and abundance of oil-degrading taxa in the phycosphere of the three species parallels their oil resistance, yet the absence of the phycosphere does not significantly alter their resistance. This suggests that both the oil resistance and the phycosphere communities are characteristics of the algal species. Previous studies showed that phytoplankton release strain-dependent organic compounds (Becker et al., 2014), leading to host-specific bacterial community structures (Bagatini et al., 2014). The occurrence of oil-degrading bacteria in the dinoflagellates phycosphere could be explained by their high content of triacylglycerols (Harrington et al., 1970; Fuentes-Grünewald et al., 2009), a precursor of biodiesel (Vasudevan and Briggs, 2008). Higher resolution of associated-bacteria community function and the interaction with their host could be obtained by combining “omics” analyses with this taxonomic information. Moreover, comparative transcriptomics of the xenic and axenic cultures might reveal a more important role of the phycosphere into the phytoplankton oil response.

The different phytoplankton oil tolerances have implications for environmental modifications following oil spills. For instance, an ecosystem dominated by small diatoms would face a group shift after an oil spill, which could result in an important decrease of carbon fixation, as diatoms are often major contributors to carbon fixation. In contrast, an oil spill in an ecosystem dominated by dinoflagellates may only result in a species shift. The global function could then be maintained, except if toxic dinoflagellate species come to dominate the ecosystem.

## Author Contributions

TS and DE contributed to conception and design of the study. TS carried out the experimental work and wrote the first draft of the manuscript. All authors contributed to manuscript revision, read, and approved the submitted version.

### Conflict of Interest Statement

The authors declare that the research was conducted in the absence of any commercial or financial relationships that could be construed as a potential conflict of interest.

## Supplementary Material

Supplementary Figure S1 Free-living (FL) and phytoplankton-attached (PA) bacteria taxonomic distribution at the family level in *Amphidinium carterae, Peridinium sociale* and *Skeletonema* sp. cultures. Labels are the treatments: CT0 for control sampled at time 0, CTF for control sampled at final time point; D for dispersant, O for oil, and OD for dispersed oil all at final time point. OTUs occurring at less than 0.001% were pool together under the label Other. The genera of known oil-degrading bacteria are annotated with an asterisk including the genus in brackets.

Supplementary Table 1 *P*-values of the Student tests between X and AX phytoplankton physiological parameters. Treatments are C for control, D for dispersant alone, O for crude oil, OD for dispersed crude oil. Significant *p*-values are indicated by stars: < 0.05 (^*^); < 0.01 (^**^); < 0.001 (^***^). *P*-values are in bold when the parameter is significantly higher in the X than AX cultures.

Supplementary Table 2 LMM *post-hoc* Dunnett test values and *p*-values of FL and PA bacterial abundance and ANOVA *post-hoc* Dunnett test values and *p*-values of aminopeptidase, glucosidase and lipase extracellular activities between control and each treatment for each phytoplankton species. A positive test value indicates a higher abundance or activity in the treatment than in the control and reciprocally, a negative test value indicates a lower abundance or activity in the treatment than in the control. Treatments are C for control, D for dispersant alone, O for crude oil, OD for dispersed crude oil. Significant *p*-values are indicated by stars: < 0.05 (^*^); < 0.01 (^**^); < 0.001 (^***^).

Supplementary Table 3 SIMPER analyses of the OTUs collectively accounting for at least 50% of the total dissimilarity between the bacterial communities of *Amphidinium carterae* and *Peridinium sociale* cultures (top), of *Amphidinium carterae* and *Skeletonema* sp. cultures (middle) and *Peridinium sociale and Skeletonema* sp. cultures (bottom). Columns 2 and 3: average percent of total reads of the OTU in each species compared. Contribution: percentage of the dissimilarity explained by each OTU. *P* value obtained after 999 iterations of the SIMPER test.

Supplementary Table 4 Results of the collinearity tests between the selected variables for the forward selection tests, and their collinear variables (noted co-variables). Significant variables indicated by the forward selection test for the CCA are indicated in bold. Collinearity and forward selection tests were done for each phytoplankton species (*Amphidinium carterae, Peridinium sociale* and *Skeletonema* sp.) and for the three species together (noted All).

Supplementary Table 5 Detailed results of the Indicator Species Analysis of each significant cluster identified by PCoA (Figure 4A–C) and ANOSIM, for *Amphidinium carterae, Peridinium sociale*, and *Skeletonema* sp. microbiomes. IndVal is the Indicator value index, a product of the components A and B. Component A is the probability that the sample belongs to the target cluster given the fact that the species has been found (specificity); component B is the probability of finding the species in samples belonging to the specific cluster (fidelity). *P* value obtained after 9999 iterations of the Indicator Species analysis.
