# Supplementary figures and images for: The Phytoplankton Taxon-Dependent Oil Response and Its Microbiome: Correlation but Not Causation
Source: Front Microbiol. 2019 Mar 11;10:385. doi: 10.3389/fmicb.2019.00385 (PMC6421335; doi:10.3389/fmicb.2019.00385)

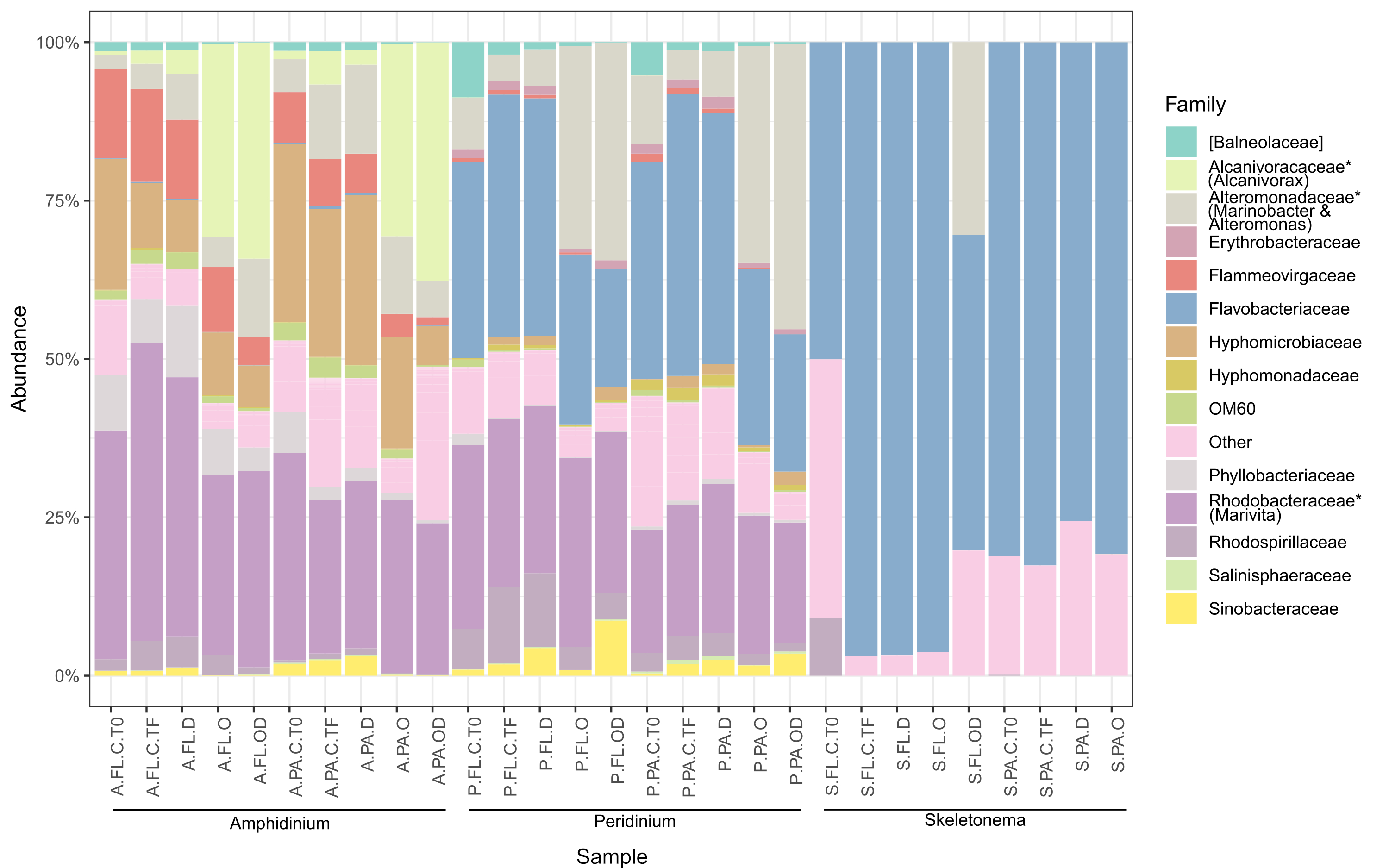

Supplement: Supplementary Figure S1 — Free-living (FL) and phytoplankton-attached (PA) bacteria taxonomic distribution at the family level in Amphidinium carterae, Peridinium sociale and Skeletonema sp. cultures. Labels are the treatments: CT0 for control sampled at time 0, CTF for control sampled at final time point; D for dispersant, O for oil, and OD for dispersed oil all at final time point. OTUs occurring at less than 0.001% were pool together under the label Other. The genera of known oil-degrading bacteria are annotated with an asterisk including the genus in brackets. [file Image_1.JPEG]
